# Cytotoxicity of α-Pyrrolidinophenones: an Impact of α-Aliphatic Side-chain Length and Changes in the Plasma Membrane Fluidity

**DOI:** 10.1007/s12640-018-9923-1

**Published:** 2018-06-27

**Authors:** Jakub Wojcieszak, Dariusz Andrzejczak, Marta Kedzierska, Katarzyna Milowska, Jolanta B. Zawilska

**Affiliations:** 1 Department of Pharmacodynamics, Medical University of Łódź, Muszynskiego 1, 90-151 Łódź, Poland; 2 Department of General Biophysics, Faculty of Biology and Environmental Protection, University of Łódź, Pomorska 141/143, 90-236 Łódź, Poland

**Keywords:** PVP, PV8, PV9, Cytotoxicity, Cell membrane fluidity

## Abstract

Pyrovalerone derivatives (α-pyrrolidinophenones) form a branch of synthetic cathinones, a second most prominent group of novel psychoactive substances. Although the toxicity of 3,4-MDPV, a progenitor of the α-pyrrolidinophenones, is well described, little is known of the potential cytotoxicity of the new members of this group entering the recreational drug market each year. The present study assesses the cytotoxicity of members of the α-pyrrolidinophenone group, i.e., α-PVP, its longer side-chain derivatives PV8 and PV9, and their 4-fluoro- and 4-methoxy-analogs, against model cell lines for the nervous system (SH-SY5Y), liver (Hep G2) and upper airway epithelium (RPMI 2650), and cardiomyocytes (H9C2(2-1)). Additionally, an impact of pyrovalerones on the fluidity of the plasma membrane, as the potential mechanism of their cytotoxicity, was examined. The longer side-chain α-pyrrolidinophenones and their fluoro- and methoxy-analogs produce more pronounced maximal cytotoxicity, with regard to mitochondrial activity and cell membrane integrity, than the five-carbon α-PVP and its substituted derivatives. The report demonstrates, for the first time, that changes of fluidity of the interior part of plasma membrane contribute to the cytotoxicity of pyrovalerone derivatives, in addition to the previously reported mechanisms. Taking into consideration our previous findings that PV8 and PV9 produce weaker psychostimulatory effects than α-PVP, the higher cytotoxicity of the new generation of pyrovalerones can pose a serious threat to abusers, as it is possible that longer-chain compounds may be taken in higher doses to obtain similar levels of stimulation.

## Introduction

Pyrovalerone derivatives (α-pyrrolidinophenones) constitute a branch of synthetic cathinones, a second most prominent group of novel psychoactive substances (NPS). Since 2008, each year has seen the introduction of a number of novel synthetic cathinone derivatives into the dynamic, clandestine NPS market, in an attempt to circumvent legal restrictions (EMCDDA 2017; Majchrzak et al. 2018; Zawilska and Wojcieszak 2017). The most remarkable examples of α-pyrrolidinophenones include 3,4-methylenedioxypyrovalerone (3,4-MDPV) and α-pyrrolidinopentiophenone (α-PVP). The new wave of pyrovalerone derivatives include compounds obtained by the modification of α-PVP structure by shortening [α-pyrrolidinopropiophenone (α-PPP), α-pyrrolidinobutiophenone (α-PBP)] or expanding an α-aliphatic side-chain [α-pyrrolidinohexanophenone (α-PHP; PV7), α-pyrrolidinoheptanophenone (α-PHPP; PV8), and α-pyrrolidinooctanophenone (α-POP; PV9)], or by the incorporation of 4-fluoro- or 4-methoxy- substituent in the phenyl ring (Zawilska and Wojcieszak 2017).

In general, pyrovalerone derivatives exert their psychostimulatory effects by elevating the extracellular level of catecholamines via blockade of the dopamine (DAT) and norepinephrine (NET) transporters responsible for reuptake of neurotransmitters, although with varying potencies (Eshleman et al. 2017; Kolanos et al. 2015; Simmler et al. 2013). The desired psychostimulatory effects include raised alertness and awareness, improved mood, impression of increased motivation, energy, and euphoria (Zawilska and Wojcieszak 2017). Contrary to previous in vitro pharmacologic characterizations (Eshleman et al. 2017), the results of a recent animal study (Wojcieszak et al. 2018) show that while longer side-chain compounds, such as PV8 and PV9, produce marked psychostimulant effect, they do so with significantly lower potency than α-PVP, which is reflected in the higher doses recommended by drug users posted on internet forums (Tripsit Factsheet n.d.; Hyperreal n.d.; PsychonautWiki n.d.), and may increase the risk of severe intoxication. The most common adverse effects caused by pyrovalerones are characteristic of the whole group of cathinones and include effects related to sympathetic stimulation, i.e., tachyarrhythmia and increased systemic blood pressure, along with neurological/psychiatric symptoms including agitation, anxiety, paranoia, seizures, restlessness, hallucinations, tremor, and bruxism (Zawilska and Wojcieszak 2017).

**Fig. 1:**
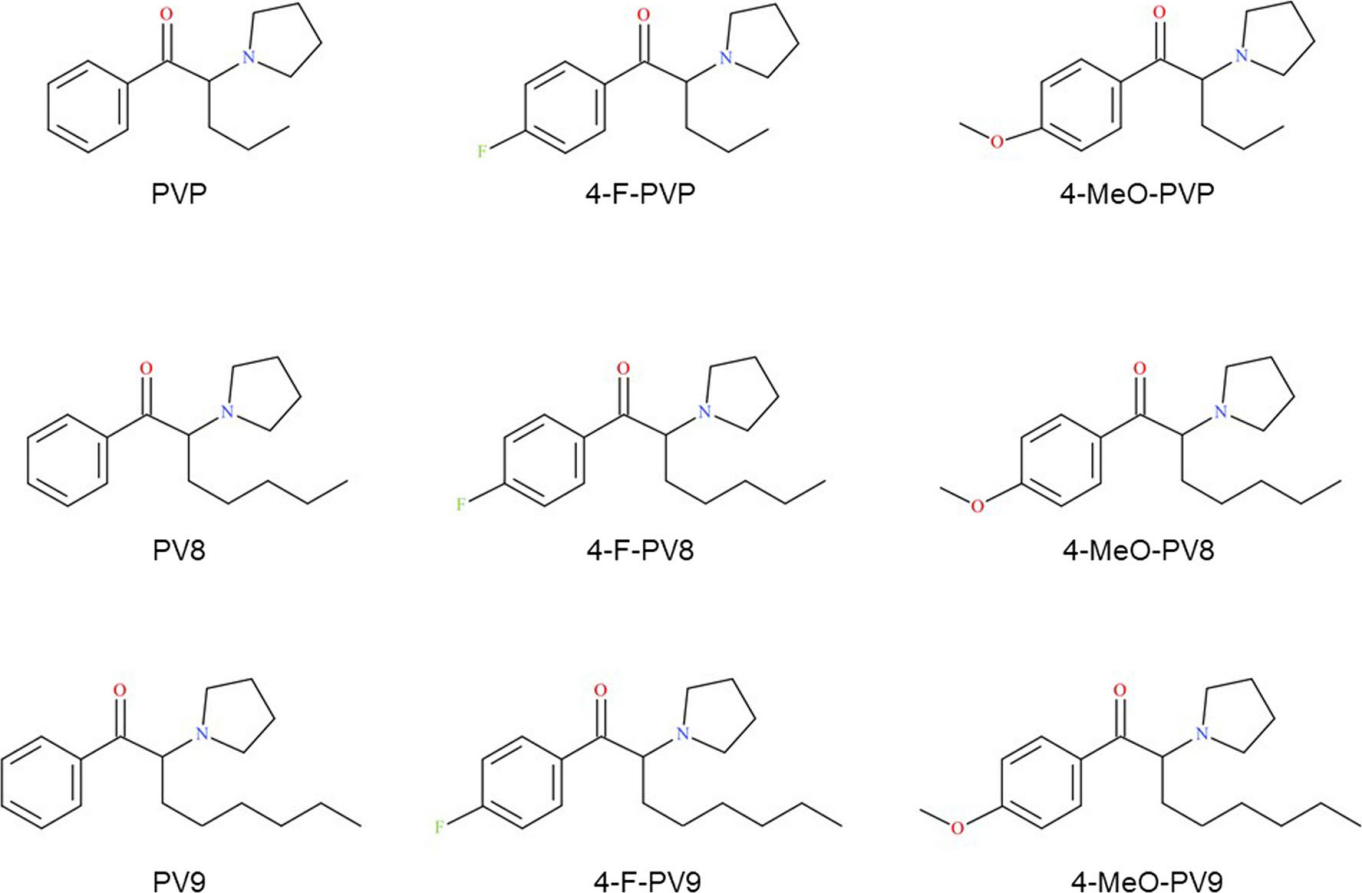
Chemical structures of α-pyrrolidinophenones used in the study

Cytotoxicity of 3,4-MDPV, a progenitor of α-pyrrolidinophenones, is well documented in hepatic and neuronal cell models, along with mechanisms of toxicity such as oxidative stress (generation of reactive oxygen and nitrogen species, depletion of reduced glutathione), disruption of mitochondrial function (depletion of ATP, decrease of mitochondrial membrane potential, inhibition of electron transport), apoptosis, and autophagy (Luethi et al. 2017; Valente et al. 2015, 2016, 2017a, b). Apart from Wojcieszak et al. (2016), we are aware of only one study, by Matsunaga et al. (2017), which investigates the toxicity of pyrovalerone derivatives with longer side-chains (PV8 and PV9), and their 4-fluoro- and 4-methoxy-analogs, on various cell lines. Similarly to 3,4-MDPV, the cytotoxicity of PV8 and PV9 was found to be based on oxidative stress followed by apoptosis. It is postulated that vascular, respiratory, and neural cells are likely to be the most sensitive to lipophilic pyrovalerones (Matsunaga et al. 2017). Elsewhere, Wojcieszak et al. (2016) report PV9 to have markedly greater cytotoxicity than that of other α-pyrrolidinophenones, such as 3,4-MDPV or pyrovalerone. This observation raises the question of whether the length of the α-carbon alkyl side-chain is an important factor determining the cytotoxicity of α-pyrrolidinophenone derivatives. Therefore, the aim of the present work was to compare the cytotoxic properties of such compounds with varying side-chain lengths: α-PVP (five carbon atoms), PV8 (seven carbon atoms), and PV9 (eight carbon atoms). Since *para-*fluoro- and *para*-methoxy- analogs of synthetic cathinones appear commonly on the NPS market, and there are case reports of intoxication with this type of compounds, *para-* substituted counterparts of α-PVP, PV8, and PV9 (see Fig. 1) were also included in the study. Experiments were performed on cell lines which reflect critical tissues for pyrovalerone toxicity, i.e., neuronal SH-SY5Y and hepatic Hep G2, or sites where extremely high concentrations of compounds may be found due to the route of their administration, i.e., RPMI 2650: a model of the upper airway system epithelium. Since no reports exist regarding the in vitro cardiotoxicity of α-pyrrolidinophenones, H9C2(2-1) rat myocardium cell line was also included in the present study. To shed more light on the possible mechanisms of cytotoxicity, changes in cellular membrane fluidity were examined using spectrofluorimetric analysis.

## Materials and Methods

### Drugs and Reagents

All the synthetic cathinones: α-pyrrolidinopentiophenone (**PVP**; 1-phenyl-2-(1-pyrrolidinyl)-1-pentanone), 4-fluoro-α-pyrrolidinopentiophenone (**4-F-PVP**; 1-(4-fluorophenyl)-2-(1-pyrrolidinyl)-1-pentanone), 4-methoxy-α-pyrrolidinopentiophenone (**4-MeO-PVP**; 1-(4-methoxyphenyl)-2-(1-pyrrolidinyl)-1-pentanone), α-pyrrolidinoheptanophenone (**PV8**; 1-phenyl-2-(1-pyrrolidinyl)-1-heptanone), 4-fluoro-α-pyrrolidinoheptanophenone (**4-F-PV8**; 1-(4-fluorophenyl)-2-(1-pyrrolidinyl)-1-heptanone), 4-methoxy-α-pyrrolidinoheptanophenone (**4-MeO-PV8**; 1-(4-methoxyphenyl)-2-(1-pyrrolidinyl)-1-heptanone), α-pyrrolidinooctanophenone (**PV9**; 1-phenyl-2-(1-pyrrolidinyl)-1-octanone), 4-fluoro-α-pyrrolidinooctanophenone (**4-F-PV9**; 1-(4-fluorophenyl)-2-(1-pyrrolidinyl)-1-octanone), and 4-methoxy-α-pyrrolidinooctanophenone (**4-MeO-PV9**; 1-(4-methoxyphenyl)-2-(1-pyrrolidinyl)-1-octanone) were purchased in the form of hydrochloride salts from Cayman Chemical (Ann Arbor, MI, USA). Cell culture reagents: DMEM, DMEM/F12 and MEM media, heat inactivated fetal bovine serum (FBS), phosphate buffered saline (PBS), Trypsin-EDTA, Non-Essential Amino Acids Solution (NEAA), penicillin, and streptomycin were purchased from Life Technologies (Warsaw, Poland). Dimethyl sulfoxide (DMSO) and MTT (3-(4,5-dimethyl-2-thiazolyl)-2,5-diphenyl-2*H*-tetrazolium bromide) were purchased from Sigma-Aldrich (Poznań, Poland). Fluorescence labels: 1-[4-(trimethylamino)phenyl]-6-phenyl-1,3,5,-hexatriene (TMA–DPH) and 1,6-diphenyl-1,3,5-hexatriene (DPH) were purchased from Sigma-Aldrich (Poznań, Poland).

For all experiments, drug solutions were prepared as follows: stock solutions were prepared in the solvent of choice (DMSO or ethanol) and 10- or 20-μl aliquots were transferred into 1.5-ml test tubes. Subsequently, organic solvent was completely removed by vacuum centrifugation at room temperature until the weight loss of samples was no longer observed. Dry pellets of the tested compounds were stored in − 20 °C, and fresh solutions in the culture medium were prepared directly before the treatment, without the use of organic solvents.

### Cell Lines

**SH-SY5Y** (ATCC® CRL-2266™), **Hep G2** (ATCC® HB-8065™), and **RPMI 2650** (ATCC® CCL-30™) cell lines were purchased from Leibniz Institute DSMZ-German Collection of Microorganisms and Cell Cultures (DSMZ, Braunschweig, Germany). **H9c2(2-1)** (ATCC® CRL-1446™) cell line was purchased from the European Collection of Cell Cultures (ECACC, Porton Down, UK).

### Cell Culture

All cell lines were cultured according to the conditions provided by the distributors. **Hep G2** and **H9c2(2-1)** cells were cultivated in DMEM, **SH-SY5Y** in DMEM/F12, and **RPMI 2650** in MEM with Earle’s salts and 1× Non-Essential Amino Acids Solution media, supplemented with 10% fetal bovine serum (FBS) and penicillin (100 U/ml)-streptomycin (100 μg/ml) at 37 °C in a humidified atmosphere enriched with 5% CO_2_.

### Cell Viability Assays

Cell viability was assessed by two complementary methods: the MTT test measuring mitochondrial red-ox activity and the LDHe assay indicating cell membrane integrity, as described previously (Wojcieszak et al. 2016). After reaching approx. 80–90% confluency, cells were harvested with 0.25% Trypsin in 1 mM EDTA and transferred into 96-well microplates for cytotoxicity assays. For the MTT assay, the cells were seeded at densities of approx. 5000 cells/well (**Hep G2**), 10,000 cells/well (**SH-SY5Y** and **RPMI 2650**), and 15,000 cells/well (**H9c2(2–1)**). For the LDHe assay, the cells were seeded at densities of approx. 10,000 cells/well (**Hep G2**), 15,000 cells/well (**H9c2(2–1)**), and 20,000 cells/well (**SH-SY5Y** and **RPMI 2650**). After overnight incubation, the complete culture medium was removed and replaced by working solutions of the tested compounds prepared in FBS-free medium. Fresh medium without FBS was used for the control group.

### MTT Test

Cell viability and mitochondrial function were measured by assessment of 3-(4,5-dimethyl-2-thiazolyl)-2,5-diphenyl-2*H*-tetrazolium bromide (MTT) reduction by mitochondrial dehydrogenases after 24- and 72-h exposure to the drugs. A solution of MTT (0.5 mg/ml) was added to the cells, and the culture was incubated for a further 3-h at 37 °C. After aspiration of culture medium, formazan crystals were dissolved in DMSO, and its absorbance was measured at 570 nm using Bio-Rad microplate reader model 680; this value being proportional to the number of cells with intact mitochondria. The mean values for each group were obtained by subtracting the mean OD of the positive control (1% (*v*/*v*) Triton-X100, added 30 min before MTT) from the value. The results are expressed as percentages of the negative control group values, these values being considered 100% viable.

### LDHe Assay

Cellular membrane integrity was assessed by measuring the activity of lactate dehydrogenase released from damaged cells into the culture medium using LDH Cytotoxicity Assay (ScienCell Research Laboratories, Carlsbad, CA, USA) following 48-h exposure to the drugs, according to the manufacturer’s instruction. Each experiment included a positive control of 1% (*v*/*v*) Triton-X100, as recommended by the manufacturer. Results are expressed as a percentage value of the positive control group, considered as 100% cytotoxicity.

### Membrane Fluidity–Fluorescence Anisotropy

Changes in the fluidity of the plasma membrane were investigated by spectrofluorimetry based on two fluorescence probes: TMA–DPH and DPH. The lipid probe DPH is located relatively deeply in the hydrocarbon interior of the lipid bilayer, while TMA–DPH is known to be located in the polar head-group region of the plasma membrane (Shinitzky and Barenholz 1978). The fluorescence anisotropy of TMA–DPH and DPH was measured at 37 °C with a Perkin-Elmer luminescence spectrometer (Model LS55B, UK): excitation was measured at 358 nm for TMA-DPH and 348 nm for DPH, while emission was measured at 428 nm for TMA-DPH and 426 nm for DPH. Prior to anisotropy measurement, Hep G2 cells (1.5 × 10^6^ cells/ml) were incubated with the tested compounds for 15 min. The final concentration of the fluorescence probe in the samples was 1 μM.

The degree of fluorescence anisotropy (*r*) was calculated automatically according to the equation:

$$ r=\left({I}_{vv}\hbox{--} {I}_{vh}\times G\right)/\left({I}_{vv}+{I}_{vh}\times G\right) $$

where *I*_*vv*_ and *I*_*vh*_ are the intensities measured with the polarization plane parallel (*I*_*vv*_*)* and perpendicular *(I*_*vh*_*)* to that of the excitation beam. *G* is a factor used to correct the polarization of the instrument and is given by the ratio of vertically to horizontally polarized emission components when the excitation light is polarized in the horizontal direction.

### Data Analysis

Data are presented as mean ± SEM from three to five independent experiments for MTT, two or three independent experiments for LDH, and three independent experiments for fluorescence anisotropy. Statistical analysis was performed using GraphPad Prism 6 (GraphPad Software, San Diego, CA, USA). The normality of distribution was checked using the Shapiro-Wilk test. The statistical analysis was performed using analysis of variance (ANOVA); the groups were compared with controls using Dunnett’s post hoc test where normal distribution was found and the Mann-Whitney test in the case of non-normal distribution. Post hoc tests were performed only in ANOVA-indicated significant effects of the treatment. Differences were considered significant when *p* < 0.05.

## Results

### Effects of PVP, 4-F-PVP, and 4-MeO-PVP on the Survival of SH-SY5Y, Hep G2, RPMI 2650, and H9c2(2-1) Cells

**Fig. 2:**
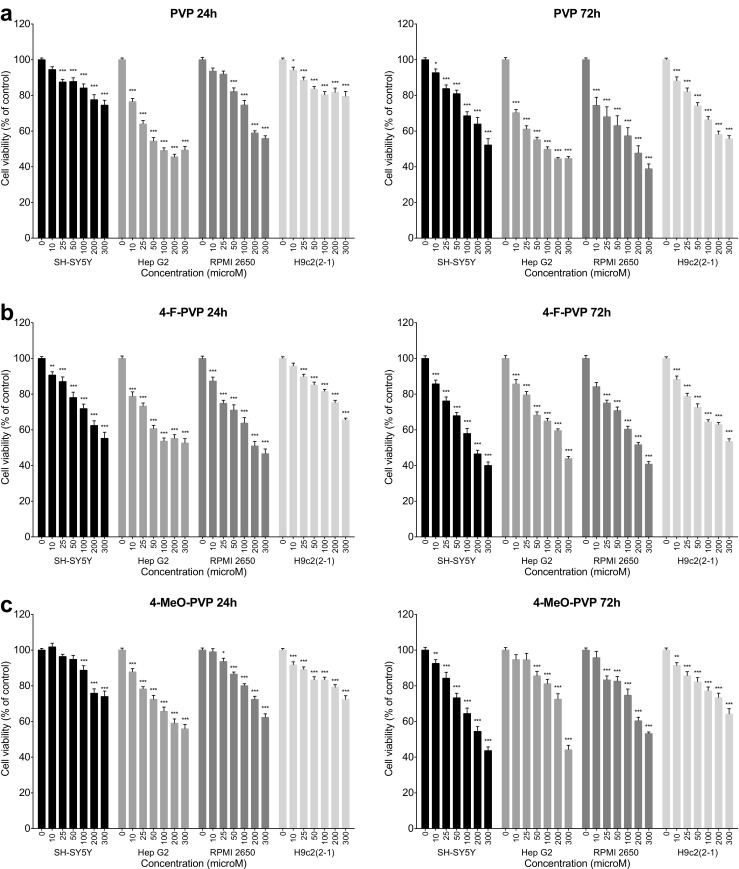
Effects of PVP (**a**), 4-F-PVP (**b**), and 4-MeO-PVP (**c**) on the viability of SH-SY5Y, Hep G2, RPMI 2650, and H9c2(2-1) cells, measured by the MTT assay. Data are mean ± SEM of 17–24 values per group and expressed as a percentage of the respective control (untreated cells). ***p < 0.001, **p < 0.01, *p < 0.05 versus control group

PVP and its fluoro- and methoxy-substituted derivatives produced moderate and concentration- and time-dependent decline in the viability of SH-SY5Y, Hep G2, RPMI 2650, and H9c2(2-1) cells measured as mitochondrial activity. The observed effects varied among the studied cell lines and the tested compounds (Fig. 2).

After 24-h incubation PVP caused significant reductions in the survival of SH-SY5Y (25–300 μM), Hep G2 (10–300 μM), RPMI 2650 (50–300 μM), and H9c2(2-1) (10–300 μM) cell lines. At a concentration of 300 μM, PVP produced a decrease in the viability of SH-SY5Y, Hep G2, RPMI 2650, and H9c2(2-1) cells by, respectively, 25, 51, 44, and 21% of control values. The cytotoxic activity of PVP was potentiated by extension of the incubation time to 72 h. Exposure of cells to 300 μM of PVP reduced their viability by 48% (SH-SY5Y), 55% (Hep G2), 61% (RPMI 2650), and 44% (H9c2(2-1)) of the respective control values (Fig. 2a).

Exposure to 4-F-PVP for 24 h led to a marked, concentration-dependent reduction in the survival of SH-SY5Y (10–300 μM), Hep G2 (10–300 μM), RPMI 2650 (10–300 μM), and H9c2(2-1) (25–300 μM) cell lines. The greatest reduction in the viability, i.e., by 53% of the control value, was observed in RPMI 2650 cells, while reductions by 45, 47, and 45% of control values were observed in SH-SY5Y, Hep G2, and H9c2(2-1) cells, respectively (Fig. 2b). Incubation of cells with 4-F-PVP for 72 h resulted in a marked, concentration-dependent decline in the survival of all cell lines, with the maximal effect at the similar level in SH-SY5Y (reduction by 60%), Hep G2 (reduction by 54%), RPMI 2650 (reduction by 59%), and H9c2(2-1) (reduction by 46%) (Fig. 2b).

Twenty four-hour treatment with 4-MeO-PVP led to a significant, concentration-dependent decrease of the viability of SH-SY5Y (100–300 μM), Hep G2 (10–300 μM), RPMI 2650 (25–300 μM), and H9c2(2-1) (10–300 μM) cells. The most pronounced reduction in the survival, i.e., by 44% of a control value, was observed in Hep G2 cells; among the other cell lines, SH-SY5Y demonstrated a 26% decrease, RPMI 2650 a 38% decrease, and H9c2(2-1) − 28%. Exposure of cells to 4-MeO-PVP for 72 h resulted in a significant decline of the viability of SH-SY5Y, H9c2(2-1) (both at 10–300 μM), RPMI 2650 (25–300 μM), and Hep G2 (50–300 μM) cells: the viability of the SH-SY5Y and Hep G2 cell lines fell to 56% of control values, RPMI 2650 to 47%, and H9c2(2-1) to 36% (Fig. 2c).

**Fig. 3:**
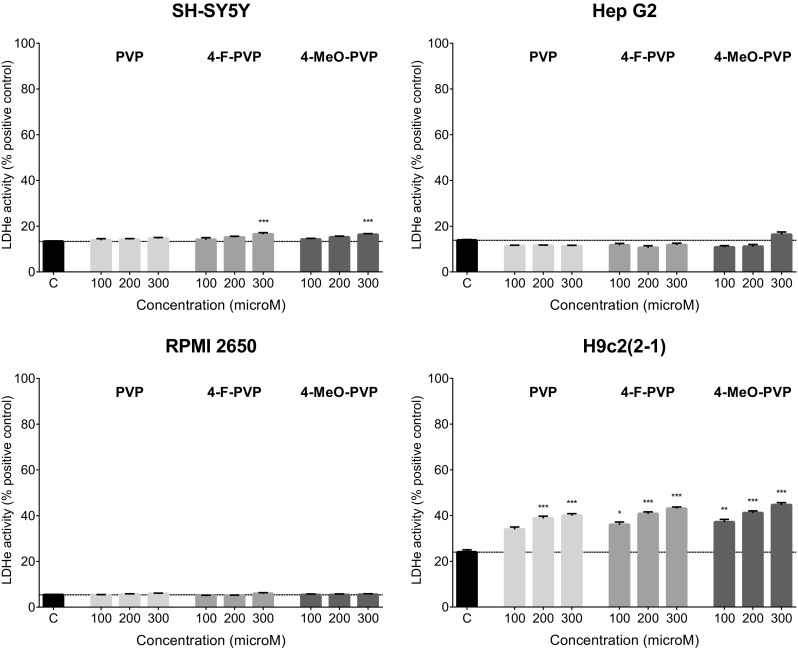
Effects of PVP, 4-F-PVP, and 4-MeO-PVP on the cell membrane integrity of SH-SY5Y, Hep G2, RPMI 2650, and H9c2(2-1) cells, measured by the LDH assay. Data are mean ± SEM of 10–15 values per group and expressed as a percentage of the positive control. ***p < 0.001, **p < 0.01, *p < 0.05 versus negative control group

PVP and its substituted counterparts produced only benign damage to the cell membranes after 48 h incubation (Fig. 3). The damage was significant only in SH-SY5Y and H9c2(2-1) cells. In SH-SY5Y neuroblasts, only 4-F-PVP and 4-MeO-PVP used at 300 μM caused a slight elevation of extracellular LDH activity. In H9c2(2-1) cells the effect was more pronounced and significant at 200 and 300 μM for PVP and 100–300 μM for 4-F-PVP and 4-MeO-PVP. The strongest effect was observed at 300 μM of 4-F-PVP and 4-MeO-PVP, being 43 and 45% of positive control cytotoxicity, respectively (Fig. 3).

### Effects of PV8, 4-F-PV8, and 4-MeO-PV8 on the Survival of SH-SY5Y, Hep G2, RPMI 2650, and H9c2(2-1) Cells

**Fig. 4:**
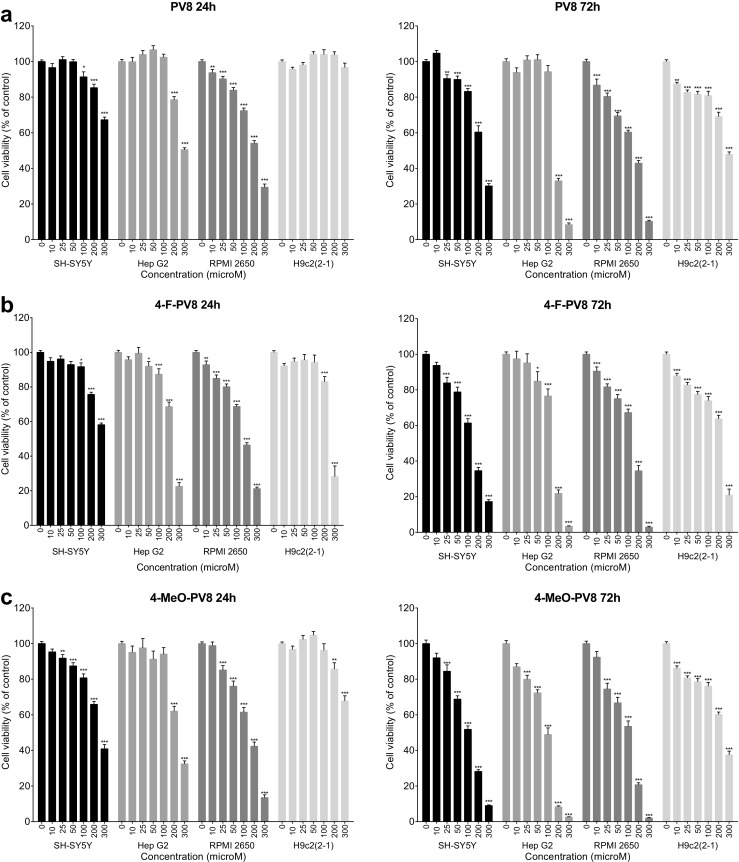
Effects of PV8 (**a**), 4-F-PV8 (**b**), and 4-MeO-PV8 (**c**) on the viability of SH-SY5Y, Hep G2, RPMI 2650, and H9c2(2-1) cells, measured by the MTT assay. Data are mean ± SEM of 15–24 values per group and expressed as a percentage of the respective control (untreated cells). ***p < 0.001, **p < 0.01, *p < 0.05 versus control group

PV8 and its substituted analogs caused loss of the viability in all tested cell lines, albeit with varying potency (Fig. 4). Exposition to PV8 for 24 h resulted in a concentration-dependent cytotoxicity in SH-SY5Y (100–300 μM), Hep G2 (200 and 300 μM), and RPMI 2650 (10–300 μM), but not H9c2(2-1) cells. The greatest decline in the viability was observed in RPMI 2650 cells (by 71% of the control value), while the most pronounced cytotoxic effect observed in SH-SY5Y and Hep G2 cells was associated with the viability drop by 33 and 50%, respectively (Fig. 4a). Extending incubation time to 72 h resulted in the strongest effects and cytotoxicity in H9c2(2-1) cells. Significant loss of the survival was observed at concentrations of 10–300 μM (H9c2(2-1) and RPMI 2650), 25–300 μM (SH-SY5Y), 200 and 300 μM (Hep G2). The greatest cytotoxicity was associated with a reduction of cell viability by 70% (SH-SY5Y), 92% (Hep G2), 90% (RPMI 2650), and 53% (H9c2(2-1)) (Fig. 4a).

Substituted analogs differ from native PV8 as they affect H9c2(2-1) cell viability even after 24 h. 4-F-PV8 applied for 24 h markedly reduced the viability of SH-SY5Y (100–300 μM), Hep G2 (50–300 μM), RPMI 2650 (10–300 μM), and H9c2(2-1) (200 and 300 μM) cell lines, with the greatest reduction by 42% (SH-SY5Y), 77% (Hep G2), 79% (RPMI 2650), and 72% (H9c2(2-1)) (Fig. 4b). Extending incubation time to 72 h augmented the maximal cytotoxic effect of 4-F-PV8. Cell viability was significantly decreased in SH-SY5Y (25–300 μM; maximal reduction by 83%), Hep G2 (50–300 μM; maximal reduction by 97%), RPMI 2650 (10–300 μM; maximal reduction by 97%), and H9c2(2-1) cells (10–300 μM; maximal reduction by 79%) (Fig. 4b).

Similarly, after 24-h incubation, 4-MeO-PV8 decreased the viability of all tested cell lines (25–300 μM for SH-SY5Y and RPMI 2650 cells, 200 and 300 μM for Hep G2 and H9c2(2-1) cells), with the greatest effect being 59% reduction of the survival for SH-SY5Y, 68% for Hep G2, 87% for RPMI 2650, and 33% for H9c2(2-1). Extending incubation time to 72 h increased the cytotoxicity at 300 μM, leading to the decrease of the viability by 91% for SH-SY5Y, 97% for Hep G2, 98% for RPMI 2650, and 63% for H9c2(2-1). Moreover, a broader concentration range was found to elicit a significant drop in the viability for the Hep G2 (25–300 μM) and H9c2(2-1) (10–300 μM) cell lines, compared to 24-h exposure (Fg. 4c).

**Fig. 5:**
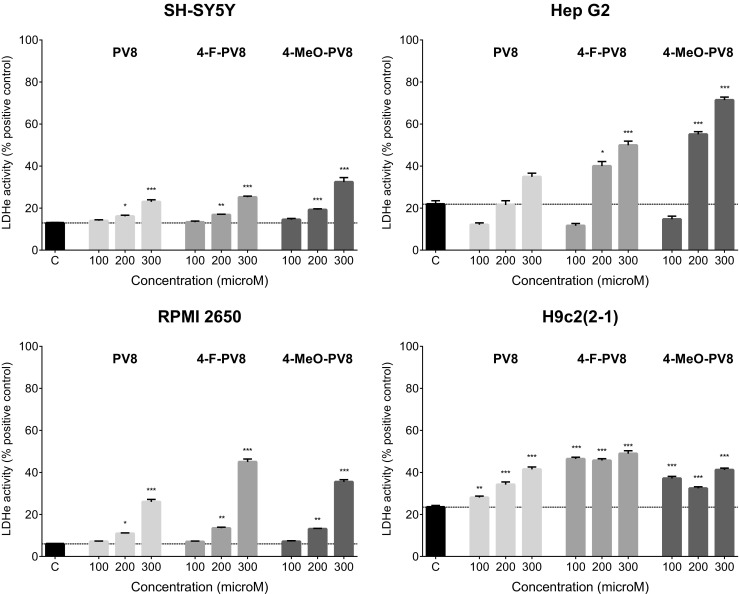
Effects of PV8, 4-F-PV8, and 4-MeO-PV8 on the cell membrane integrity of SH-SY5Y, Hep G2, RPMI 2650, and H9c2(2-1) cells, measured by the LDH assay. Data are mean ± SEM of 10–15 values per group and expressed as a percentage of the positive control. ***p < 0.001, **p < 0.01, *p < 0.05 versus negative control group

PV8, 4-F-PV8, and 4-MeO-PV8 inflicted significant damage to the membranes of SH-SY5Y and RPMI 2650 cells at 200 and 300 μM, and to H9c2(2-1) cells when applied at 100 to 300 μM. Moreover, 4-F-PV8 and 4-MeO-PV8, but not PV8, disrupted Hep G2 cell membranes at 200 and 300 μM. The most pronounced cytotoxicity was observed after treatment of Hep G2 cells with 4-MeO-PV8 (71% of positive control toxicity) (Fig. 5).

### Effects of PV9, 4-F-PV9, and 4-MeO-PV9 on the Survival of SH-SY5Y, Hep G2, RPMI 2650, and H9c2(2-1) Cells

**Fig. 6:**
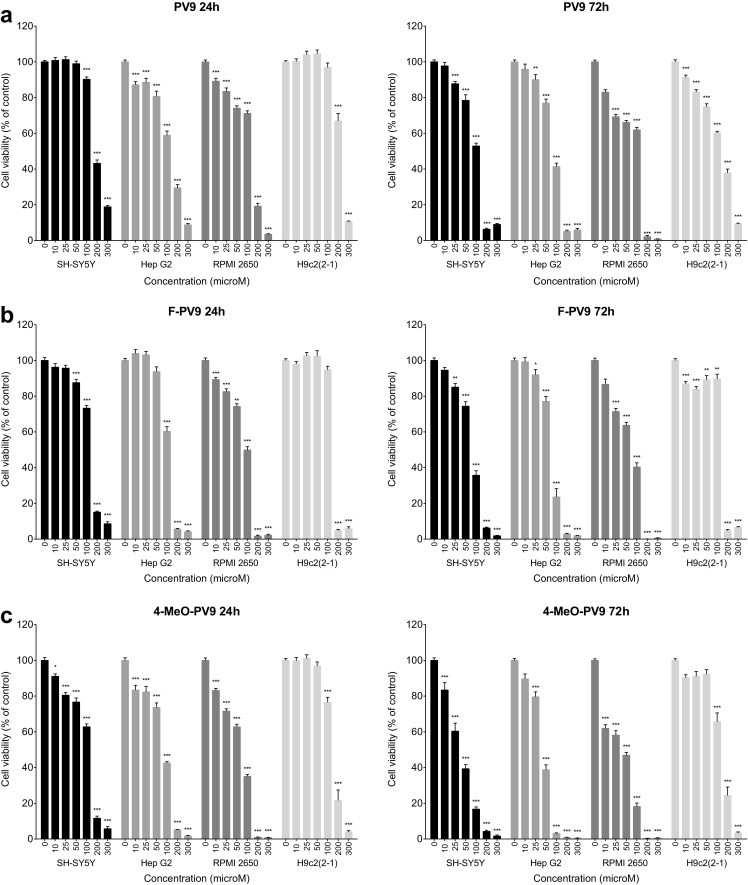
Effects of PV9 (**a**), 4-F-PV9 (**b**), and 4-MeO-PV9 (**c**) on the viability of SH-SY5Y, Hep G2, RPMI 2650, and H9c2(2-1) cells, measured by the MTT assay. Data are mean ± SEM of 17–30 values per group and expressed as a percentage of the respective control (untreated cells). ***p < 0.001, **p < 0.01, *p < 0.05 versus control group

PV9 and its substituted analogs produced significant cytotoxicity in all analyzed cell lines, with profound effects observed after 24 h incubation at concentrations of 200 and 300 μM (Fig. 6). Treatment with PV9 for 24 h caused a significant decrease in the survival of Hep G2 and RPMI 2650 (10–300 μM), SH-SY5Y (100–300 μM), and H9c2(2-1) (200 and 300 μM) cells. Cell viability was reduced to below 30% of the control group values by 200 and 300 μM PV9 in Hep G2 (max. reduction by 91%) and RPMI 2650 cells (max. reduction by 96%), and by 300 μM PV9 in SH-SY5Y (max. reduction by 81%) and H9c2(2-1) cells (max. reduction by 89%) (Fig. 6a). After extending incubation time to 72 h, PV9 caused almost complete loss of viable SH-SY5Y (max. reduction by 94%), Hep G2 (max. reduction by 95%), and RPMI 2650 cells (max. reduction by 99%) when applied at 200 and 300 μM, and a 91% reduction of H9c2(2-1) when applied at 300 μM. A significant reduction of viable cells was observed in concentrations of 10–300 μM in H9c2(2-1) cells, and at concentrations ranging from 25 to 300 μM in SH-SY5Y, Hep G2, and RPMI 2650 cells (Fig. 6a).

4-F-PV9 induced severe cytotoxicity at 200 and 300 μM in all cell lines, resulting in a decrease of the viability to below 15% of control group values after 24 h of incubation (max. reduction of 91% in SH-SY5Y, 96% in Hep G2, 98% in RPMI 2650, and 95% in H9c2(2-1) cells), and below 7% after 72 h (max. reduction of 98% in SH-SY5Y, 98% in Hep G2, 100% in RPMI 2650, and 95% in H9c2(2-1) cells). Significant reduction of cell viability was observed in RPMI 2650 cells at concentrations of 10 to 300 μM, in SH-SY5Y cells between 50 and 300 μM, in Hep G2 cells between 100 and 300 μM, and in H9c2(2-1) cells at 200 and 300 μM after 24-h incubation. Significant reductions were also seen in H9c2(2-1) cells between 10 and 300 μM, and in SH-SY5Y, Hep G2, and RPMI 2650 cells between 25 and 300 μM after 72-h incubation (Fig. 6b).

4-MeO-PV9 caused severe cytotoxicity in SH-SY5Y, Hep G2, and RPMI 2650 cells when administered at concentrations of 200 and 300 μM, resulting in at least a 88% decrease of the viability after 24 h (max. reduction by 94% in SH-SY5Y, 98% in Hep G2, and 99% in RPMI 2650), and at least a 95% reduction after 72 h (max. reduction of 98% in SH-SY5Y, 99% in Hep G2, and 100% in RPMI 2650). In H9c2(2-1) cells, the viability was reduced by 76–78% at 200 μM and by approximately 96% at 300 μM, irrespective of the incubation time. In the concentration range of 10–300 μM, significant cytotoxic effects were observed in SH-SY5Y, Hep G2, and RPMI 2650 cells after 24-h incubation, and in SH-SY5Y and RPMI 2650 cells after 72-h incubation. However, at 25–300 μM, the viability of Hep G2 cells was affected only after 72-h incubation. In H9c2(2-1) cells, significant effects were observed at 100–300 μM, irrespective of the incubation time (Fig. 6c).

**Fig. 7:**
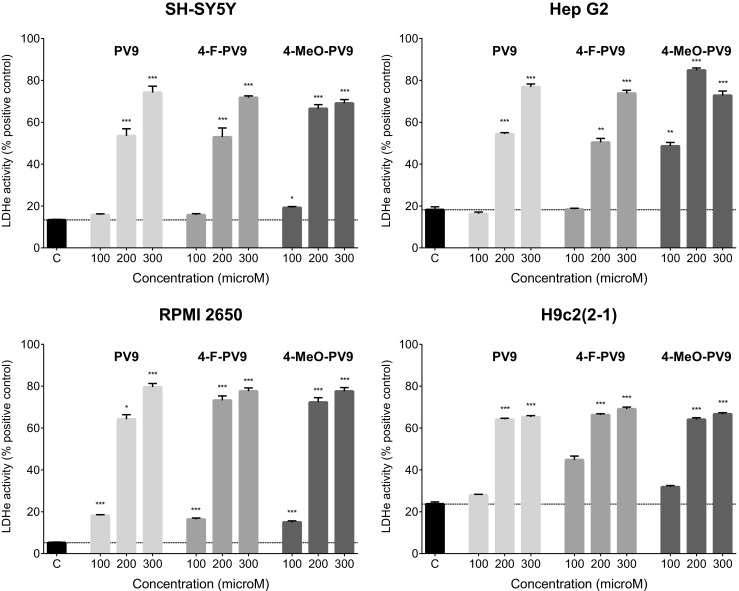
Effects of PV9, 4-F-PV9, and 4-MeO-PV9 on the cell membrane integrity of SH-SY5Y, Hep G2, RPMI 2650, and H9c2(2-1) cells, measured by the LDH assay. Data are mean ± SEM of 10–15 values per group and expressed as a percentage of the positive control. ***p < 0.001, **p < 0.01, *p < 0.05 versus negative control group

PV9 and its substituted analogs caused profound disruption of cell membranes in all assessed cell lines (Fig. 7). The effect was always significant at 200 and 300 μM for all drugs and cell lines; in addition, the membrane integrity of RPMI 2650 cells was also significantly affected by all drugs at 100 μM. Moreover, 4-MeO-PV9 also damaged the membranes of SH-SY5Y and Hep G2 cells when administered at 100 μM. All PV9 analogs produced a maximal effect of at least 70% of the positive control group in SH-SY5Y, Hep G2, and RPMI 2650 cells, while the maximal effect of PV9 derivatives in H9c2(2-1) cells always exceeded 65% of the positive control (Fig. 7).

### Effects of PVP, PV8, and PV9 on Membrane Fluidity

**Fig. 8:**
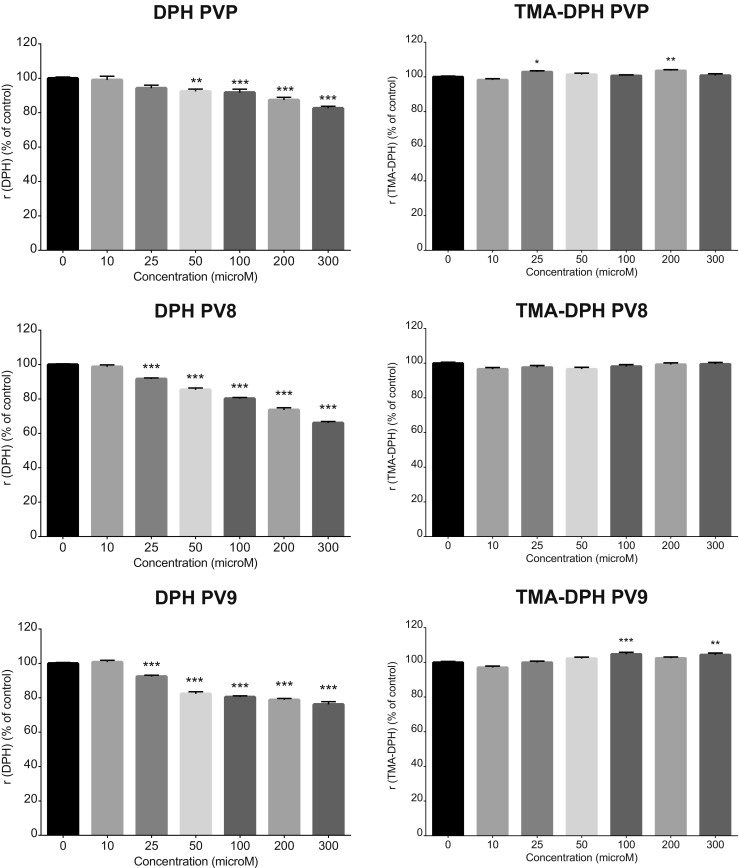
Effects of PVP, PV8, and PV9 on the plasma membrane fluidity of Hep G2 cells, measured by fluorescence anisotropy. Data are mean ± SEM of 16–18 values per group and expressed as a percentage of the respective control (untreated cells). ***p < 0.001, **p < 0.01, *p < 0.05 versus control group

Exposure of Hep G2 cells to PVP, PV8, and PV9 for 15 min significantly lowered the fluorescence anisotropy of the DPH probe, which is negatively related to the fluidity of the inner part of the cell membrane. Statistically significant increases in membrane fluidity were observed in the concentration range from 50 to 300 μM for PVP and 25 to 300 μM for PV8 and PV9, while the most pronounced effect was observed after treatment with PV8 (300 μM) (Fig. 8). However, neither PVP, PV8, or PV9 lowered the fluorescence anisotropy of the TMA-DPH probe, which reflects the fluidity of the polar head-group portion of the cell membrane (Fig. 8).

## Discussion

Our findings demonstrate that α-pyrrolidinophenones decrease the viability of cell lines derived from neuroblasts, hepatic epithelium, upper airway epithelium, and cardiomyoblasts in a concentration- and time-dependent manner. The length of the α-aliphatic side-chain seems to be the most important factor determining the maximal cytotoxic effect, i.e., neither α-PVP, with five carbon atoms, nor its substituted analogs lowered cell viability to considerably below 40% of the control values in all tested cell lines. On the other hand, the incubation of cells with PV8 and its analogs, containing an additional two carbon atoms, reduced cell viability by a maximum of 70–80% after 24 h, and by more than 90% after 72 h. Further elongation of the side-chain increased the cytotoxic activity, as observed in the tests on PV9 and its substituted analogs. Incubation for 72 h with the highest tested doses (200 and 300 μM) resulted in almost complete extinction of cell viability in all but the H9C2(2-1) cell lines.

Our findings are in line with those published by Matsunaga et al. (2017), demonstrating that cytotoxicity of pyrovalerones increases with the elongation of the α-carbon side-chain. Longer side-chain pyrovalerones, i.e., PV8 and PV9, are known to produce a weaker stimulatory effect in mice and less prominent elevation of extracellular dopamine levels in the mouse striatum than PVP (Wojcieszak et al. 2018), which in turn is reflected in the use of higher doses of PV8 and PV9 than PVP (Tripsit Factsheet n.d.; Hyperreal n.d.; PsychonautWiki n.d.). Importantly, our results suggest that the risk of intoxication with pyrovalerones, resulting from their cytotoxic properties, could be positively related to the length of their aliphatic side-chain.

Results obtained using LDH assay further confirm the impact of the side-chain length on the cytotoxicity of pyrrolidinophenones. PVP and its analogs caused only benign disruption of SH-SY5Y and H9C2(2-1) cell membranes, while PV8 and PV9, and their substituted derivatives, evoked marked damages. Our findings, based on fluorescence anisotropy, demonstrate for the first time that the disruption of the plasma membrane fluidity observed after a short, 15 min exposure is a significant factor underlying the cytotoxicity of pyrrolidinophenones, in addition to the previously reported induction of oxidative stress, activation of caspases leading to apoptosis, mitochondrial disruptions, and autophagy (Luethi et al. 2017; Valente et al. 2015, 2016, 2017a, b). Importantly, the more lipophilic PV8 and PV9 evoked changes in the membrane fluidity across a broader concentration range than PVP, an observation that is in line with the fact that disturbances were found in the internal, highly lipophilic part of the membrane but not in the external polar head-groups.

LDH assay revealed marginal disruption of cell membrane integrity after treatment with PVP and its substituted analogs in all but the H9c2(2-1) cells, which suggests that changes in the membrane fluidity may be reversible. This hypothesis can be further supported by the fact that in the case of PVP and its analogs, the maximal cytotoxic effect measured by the MTT assay was not significantly augmented by prolongation of the incubation time from 24 to 72 h. On the other hand, PV8, PV9, and their analogs exhibited markedly higher cytotoxic potential after longer incubation time. Therefore, it can be conjectured that the administration of more lipophilic substances may be associated with irreversible changes in the membrane fluidity, leading to poration of the membrane and leakage of its cell content, as revealed by LDH assay, finally resulting in cell death.

Drug concentrations used in this in vitro study, reaching 300 μM, exceed those normally found in the blood obtained from intoxicated patients and during autopsies (Kudo et al. 2015; Marinetti and Antonides 2013). However, as discussed in our previous work (Wojcieszak et al. 2016), organs such as the liver, brain, and upper airway epithelium can be exposed to significantly higher local drug concentrations than those measured in blood. Moreover, it is noteworthy that immortalized cancer cell lines, which are a convenient model for in vitro studies, can be more resistant to cytotoxicity, and therefore, cell damage can be observed in concentrations higher than in normal cells in vivo (den Hollander et al. 2014; Wojcieszak et al. 2016). It is worth noting that concentrations used in the present study are within the range of concentrations usually used in the in vitro assessment of pyrovalerones’ and other synthetic canthinones’ cytotoxicity, and which frequently exceed 1000 μM, with the usual incubation time of 24–48 h (den Hollander et al. 2014, 2015; Luethi et al. 2017; Valente et al. 2015, 2016, 2017a, b). Long incubation times were applied in order to show whether the cytotoxicity of studied compounds increase with time, which is relevant since the common abuse pattern of synthetic cathinones includes long sessions during which multiple doses are administered (Zawilska and Wojcieszak 2013).

## Conclusions

This study confirms that pyrovalerone cathinones are endowed with the prominent cytotoxicity. The maximal cytotoxic effect increases with the elongation of the α-aliphatic side-chain, which can cause major health problems, as longer-chain substances produce less pronounced stimulatory effects and hence are used in higher doses. Additionally, the presented findings implicate the presence of disturbances in the plasma membrane fluidity as another important factor underlying the cytotoxicity of α-pyrrolidinophenones.
